# Neural and Molecular Features on Charcot-Marie-Tooth Disease Plasticity and Therapy

**DOI:** 10.1155/2012/171636

**Published:** 2012-06-13

**Authors:** Paula Juárez, Francesc Palau

**Affiliations:** ^1^Unidad de Genética y Medicina Molecular, Instituto de Biomedicina de Valencia, Consejo Superior de Investigaciones Científicas (CSIC), Jaume Roig 11, 46010 Valencia, Spain; ^2^CIBER de Enfermedades Raras (CIBERER), ISCIII, 46010 Valencia, Spain; ^3^Faculty of Medicine, University of Castilla-La Mancha, 13071 Ciudad Real, Spain

## Abstract

In the peripheral nervous system disorders plasticity is related to changes on the axon and Schwann cell biology, and the synaptic formations and connections, which could be also a focus for therapeutic research. Charcot-Marie-Tooth disease (CMT) represents a large group of inherited peripheral neuropathies that involve mainly both motor and sensory nerves and induce muscular atrophy and weakness. Genetic analysis has identified several pathways and molecular mechanisms involving myelin structure and proper nerve myelination, transcriptional regulation, protein turnover, vesicle trafficking, axonal transport and mitochondrial dynamics. These pathogenic mechanisms affect the continuous signaling and dialogue between the Schwann cell and the axon, having as final result the loss of myelin and nerve maintenance; however, some late onset axonal CMT neuropathies are a consequence of Schwann cell specific changes not affecting myelin. Comprehension of molecular pathways involved in Schwann cell-axonal interactions is likely not only to increase the understanding of nerve biology but also to identify the molecular targets and cell pathways to design novel therapeutic approaches for inherited neuropathies but also for most common peripheral neuropathies. These approaches should improve the plasticity of the synaptic connections at the neuromuscular junction and regenerate cell viability based on improving myelin and axon interaction.

## 1. Introduction

Charcot-Marie-Tooth disease (CMT) is a clinical and genetic heterogeneous group of inherited motor and sensory peripheral neuropathies (HMSN) that affect 17–40 per 100,000 inhabitants [1, 2]. Mendelian segregation in families may follow either autosomal dominant, autosomal recessive, or X-linked patterns. Autosomal recessive forms are described more frequently in specific populations and geographical areas such as the Mediterranean basin. Molecular genetic studies and positional cloning, and more recently exome sequencing approaches, have unraveled a wide number of genes involved in the etiology of CMT disease [3–5]. Molecular genetic studies have been very successful for defining the gene nosology and classification of inherited peripheral neuropathies; more than 40 genes have so far been identified to be associated with CMT and related neuropathies, including rare clinical variants (http://www.molgen.ua.ac.be/CMTmutations/). As an immediate consequence, genetic testing has become an important tool in clinical practice of CMT, and patients and families have been beneficiated of a more specific genetic counseling. CMT is caused by mutations in genes that encode proteins with different locations, including compact and noncompact myelin, Schwann cells, and axons, and that are involved in very different functions, which include compaction and maintenance of myelin, transport through myelin, transcription regulation associated with myelination, cell signaling, cytoskeleton formation, axonal transport, mitochondrial dynamics and metabolism, vesicle and endosomal trafficking, and chaperones. Whatever the metabolic or structural defect that primarily affects the myelin or the axon, the final common pathway in peripheral neuropathies is represented by an axonal degenerative process that, in most cases, mainly involves the largest and longest fibers [6–8]. Signals from axons determine whether or not a Schwann cell will alter its phenotype and make myelin. Alternatively, Schwann cell abnormalities may induce axonal degeneration with or without demyelination. Progress has been made toward understanding how particular mutations cause disease, but pathogenic mechanisms remain largely unknown. 

The PNS is a complex network of myelinated and nonmyelinated nerves of varying diameters. A myelinated nerve fiber consists of a single continuous neuronal process, the axon, surrounded along the outside by serially arranged Schwann cells, which enwrap the associated axon with their cell membrane in a multilayered specialized structure, the myelin sheath. During development, the acquisition of a myelinating phenotype by the Schwann cell appears to be in response to as yet not understood cues from the axons [9]. When the Schwann cell establishes a one-to-one association with an axon at the promyelinating stage of its development, the program of myelination is started and becomes a myelinating Schwann cell. In contrast, Schwann cells that do not establish this relationship with an axon do not activate the program of myelin gene expression and become nonmyelinating Schwann cells [10]. Interestingly, this decision process is directed by the axons, as maintenance of myelin depends on axon and axon integrity. Other example of the influence of the axons on Schwann cells is the establishment of an electrically insulate node of Ranvier. Numerous molecules mediate specific aspects of the interactions between peripheral axons and Schwann cells [11] including MAG, p75, IGF1, integrins, and TGF-*β*. Neuregulin 1 (Nrg1) and its receptors, the ErbB receptors tyrosine kinases, have emerged as key regulators of axon-Schwann cell interactions at every stage. Spinal cord motoneurons, dorsal root ganglia (DRG) sensory neurons, and autonomic neurons express Nrg1 [12], and Schwann cell express both ErbB2 and ErbB3 receptors [13]. In addition to its previously known roles in proliferation and myelination, Nrg1 type III controls Schwann cell migration. Talbot's group have recently demonstrated that Nrg1 type III is an essential signal that controls Schwann cell migration to ensure that they are present in the correct numbers and positions in developing nerves [14]. Inherited demyelinating neuropathies provide examples of how the axons are also dependent on Schwann cells. The molecular studies on the progressive axonal degeneration seen in demyelinating CMT rodent models have demonstrated that they are likely to be the result of abnormalities in Schwann cell-axonal interactions [15–17].

## 2. CMT: Inheritance and Phenotypes of Motor and Sensory Neuropathies

**Figure 1:**
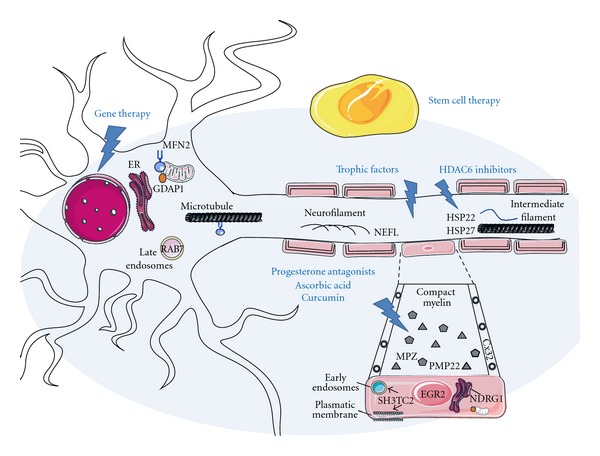
Peripheral nerve structure and cell localization of some CMT proteins at either the Schwann cell and myelin or the neuronal axon. (a) PMP22 and P0 are structural proteins located at the compact myelin and Cx32 at the noncompact myelin in the paranode (and also at the Schmidt-Lanterman incisures). Some other demyelinating CMT-associated molecules are SHT3TC2 at the plasmatic membrane and related to early endosomes and endosome recycling, the transcription factor ERG2 working in early promyelination programme, and NDRG1 that is ubiquitously expressed and has been proposed to play a role in growth arrest and cell differentiation, possibly as a signaling protein shuttling between the cytoplasm and the nucleus. Proteins mainly related to axonal CMT are associated with neurofilaments (NEFL), late endosomes (RAB7), mitochondria, endoplasmic reticulum and microtubules (MFN2 and GDAP1), or intermediate filaments (HSP22 and HSP27). (b) A ray sign indicates the main location where drugs or advanced therapies are acting. Stem cell therapy is represented as an open shadow grasping the neuron soma and axon and the Schwann cell. Trophic factors may be delivered as a drug but also by means of gene vectors or as a part of the local function of therapeutic stem cells. This figure was produced using Servier Medical Art (http://www.servier.com/servier-medical-art/powerpoint-image-bank).

CMT disease refers to peripheral neuropathies that affect both motor and sensory nerves. They are classically subdivided into “primary demyelinating” forms (CMT disease type 1 or CMT1), which are defined by a characteristic reduction of nerve conduction velocity (NCV) and segmental demyelination and remyelination, and “primary axonal” forms (CMT2) that show preservation or mild reduction of NCVs and loss of axons, namely, those of large diameter (≥8 *μ*m). As information about CMT2 is increasing, it has become evident that the distinction between CMT1 and CMT2 is less clear-cut than what was originally believed [18]. Diagnosis of CMT2 can be difficult; however, as many authors have noted, this type of CMT appears to have greater variability in its clinical presentation than CMT1. The CMT clinical phenotype is the consequence of a progressive axonal loss and degeneration affecting preferentially the longest axons, whatever the underlying primary pathogenic mechanism, either a myelinopathy and/or axonopathy [6, 7, 19]. CMT is predominantly a large-fibre neuropathy, but as sural nerve biopsies sometimes show, small fibres may be involved as well. In spite of the surprising variability of genes involved in the pathogenesis of CMT, common molecular pathways have been identified within Schwann cells and axons that cause these genetic neuropathies [5]. A review of some of most frequent forms is useful to define relevant clues to the pathogenesis of CMT and to sum up therapeutic interventions oriented to modulate the plasticity of these neuropathies (Figure 1).

### 2.1. Autosomal Dominant Demyelinating Neuropathies: CMT1

Approximately 60% of CMT patients show a predominantly demyelinating peripheral neuropathy and are classified as CMT1. The main subtype is **CMT1A **[20, 21], accounting for 40–50% of all CMT cases, which is associated with an autosomal dominant 1.4 Mb duplication on chromosome 17p11.2 that includes the peripheral myelin protein 22 gene (*PMP22*) [22, 23], a dosage-sensitive gene, expressed predominantly in the compact myelin of Schwann cells of the PNS. Less commonly, point mutations in *PMP22* have been also associated to CMT1. The mirror mutation, that is, the 1.4 Mb deletion [24, 25], and more rarely nonsense or frameshift *PMP22* mutations [26, 27] cause the myelinopathy called hereditary neuropathy with liability to pressure palsies (HNPPs) [28, 29]. Thus, duplicated or deleted of *PMP22* gives rise to demyelinating neuropathies and secondary axonal loss or abnormalities by a mechanism of gene dosage. When overexpressed in cultured cells or in transgenic mice overexpressing the human gene, PMP22 reaches late endosomes and forms protein aggregates that are ubiquitinated [30]. Removal of preexisting aggresomes formed by endogenous PMP22 is aided by autophagy [31]. A second cellular mechanism that can influence protein aggregation is the heat shock response. In proteasome-inhibited cells, overexpressed wild-type and mutant PMP22, as well as the spontaneous aggregates in neuropathic mouse nerves, recruits heat shock proteins [31]. The formation of aggresomes is a protective response of the cell that concentrates misfolded proteins in a central location to activate an autophagic response [32]. Fortun et al. propose a protective role for chaperones in preventing the accumulation of misfolded proteins. Elevated *PMP22* expression might perturb Schwann cell function by interfering with the intracellular sorting of PMP22 and other proteins, leading to overloading of the protein degradation machinery. Although demyelination is the pathological and physiological hallmark of CMT1A, the clinical signs and symptoms of this disease, progressive weakness, and sensory loss are produced by axonal degeneration [6]. The characteristic features of the PMP22 mutant Trembler and Trembler-J mice [33], particularly the minimal or abnormal myelination and reduced axonal diameter, have made them very attractive models to define the mechanisms by which defective peripheral myelination can modify axonal properties.

**CMT1B** is caused by mutations in the major myelin protein zero gene (*MPZ*), which comprises approximately 50% of myelin protein, and is necessary for both normal myelin structure and function [34, 35]. To date there are more than 150 different mutations in *MPZ* known to cause CMT1B in patients, which include missense, nonsense, small insertion/deletion, and splice site mutations. Based on clinical studies, Shy and coworkers' [36] patients fall into two distinct phenotype groups: one causing delayed motor development and marked slow nerve conduction and a second one usually associated with late-onset neuropathy, which allows developmental myelination, but eventually leads to axonal degeneration with minimal evidence of demyelination. It is difficult to make genotype-phenotype correlations because mutations in *MPZ* impair the adhesive function of myelin protein zero (P0), its subcellular trafficking, or both. Either abnormal gain-of-function effects (toxicity of misfolded protein) or reduced amounts of P0 (haploinsufficiency) could, therefore, underlie the clinical phenotype [37]. Pennuto and collaborators demonstrated that the unfolded protein response (UPR) activated by overload of misfolded proteins in the ER was responsible for demyelination in a CMT1B mouse model [38]. A recent work has identified that an increased gene dosage of *MPZ* is directly involved in the pathogenesis of human peripheral nerves [39].

### 2.2. Autosomal Dominant Axonal Neuropathies: CMT2

CMT2 has a highly heterogeneous genotype. Mutations in the mitofusin 2 gene (*MFN2*) cause CMT2A and account for about 20% of CMT2 cases. Other less frequently mutated genes are *MPZ* (CMT2J), which also causes CMT1B, and the neurofilament light chain gene (*NEFL*) [40, 41].

**CMT2A** MFN2 participates in the fusion pathway of the mitochondrial dynamics [42, 43] and is also involved in the relationship of mitochondria with endoplasmic reticulum (ER); furthermore, depletion of MFN2 causes a disruption of mitochondrial dynamics and abnormalities in Ca^2+^ homeostasis [44]. Mechanisms that have been proposed to explain the pathophysiology of CMT2A associated with MFN2 dysfunctions include a defect in mitochondrial fusion, leading to a loss of mtDNA, and impairment in oxidative phosphorylation and cell bioenergetics [45]. Current models propose that a mitochondrial transport defect could be the cause of CMT2A. Zhao et al. were the first to link axonal cargo transport dysfunction to CMT2A [46]. Based on several studies [47, 48], it is tempting to speculate that MFN2 could be part of a motor complex involved in anterograde movement of mitochondria. So far, two transgenic mouse models expressing pathogenic mutations have been generating, *Mfn*2^T105M^ [49] and *Mfn*2^R94Q^ [50]. Loss of MFN2 profoundly and selectively disrupts axonal mitochondrial transport [51], which indicates its integral role in the regulation of mitochondrial transport, and the important implications for understanding the pathophysiology of CMT2A.

To date, up to 18 neurofilament light (*NEFL*) mutations have been associated with axonal **CMT2E** [52, 53]. A conditional mouse model, carrying the P22S mutation, mimics many aspects of the human CMT2E disease, including motor disability, abnormal muscle morphology, and denervation events [54]. These results highlight the importance of the integrity of the neurofilament network for neuronal function and suggest that the disease symptoms caused by the *NEFL* ^*P*22*S*^ mutation might result from axonal transport defects rather than deleterious effects of large neurofilament aggregates.

### 2.3. X-Linked CMT

With a frequency of about 10%, **CMTX1 **[55] is the second most common inherited neuropathy. CMTX1 is genetically defined by mutations in the gene *GJB1, *which encodes the gap junction protein connexin-32 (Cx32) on the Xq13 chromosome [56]. Cx32 is localized in the noncompacted myelin sheath of large diameter fibers, forming the functional channels that allow for the rapid transport of ions and small nutrients between coupled cells [37]. Although Cx32 expression is not limited to the peripheral nervous system (it is also expressed by white matter oligodendrocytes), Cx32 mutations are associated only with CMT [57]. So far, more than 270 mutations that alter the structure of Cx32 have been reported, and most of these probably cause a partial or complete loss of function. *Cx32*-deficient mice have prominent adaxonal changes at the ultrastructural level, and a similar pathomechanism is observed in humans [58].

### 2.4. Autosomal Recessive CMT: Demyelinating CMT4 and Axonal AR-CMT2 Variants

In 2002, Baxter et al. [59] and Cuesta et al. [60] demonstrated that mutations in the ganglioside-induced differentiation-associated protein-1 (*GDAP1*) gene cause autosomal recessive CMT neuropathy. This finding was fascinating, as the two reports differed markedly with respect to the phenotypes of their families. Cuesta et al. described families who had an axonal phenotype (**AR-CMT2K**), whereas Baxter's families showed a demyelinating phenotype (**CMT4A**). Both slow and normal NCVs have been reported in patients, and many of the cases show a severe phenotype and have their onset in childhood. However, mild forms segregating as an autosomal dominant phenotype have also been reported [61]. Mutations have been described in every exon and include missense, nonsense, splicing site, short deletions, and insertion mutations. GDAP1 belongs to a glutathione S-transferase enzyme subfamily [62] that is mainly expressed in neurons [63, 64] but also in Schwann cells [65]. GDAP1 is a mitochondrial protein [63] located in the mitochondrial outer membrane (MOM) acting as a regulator of mitochondrial dynamics [65, 66]. The effect of *GDAP1 *mutations in mitodynamics seems to depend on the inheritance pattern [67]. Overexpression of GDAP1 in COS7 or HeLa cells causes mitochondrial fragmentation or fission and a substantial accumulation of mitochondria around the nucleus. Rescue experiments in *Saccharomyces cerevisiae* defective mutans for fission and fusion genes have shown that *GDAP1 *rescues the phenotype of the fission-associated gene *Fis1*. In particular, the recovery of G2/M delay suggests that both Fis1p and GDAP1 may affect the interaction of mitochondria with microtubules [68], an aspect that may relate GDAP1 to mitochondrial transport or movement in axons.

**CMT4C **neuropathy, which is caused by mutations in the *SH3TC2* gene, is the most common cause of the autosomal recessive form of demyelinating CMT. *SH3TC2* is specifically expressed in Schwann cells and is necessary for proper myelination of peripheral axons. Analysis of the murine model of CMT4C revealed that the capacity of SH3TC2-deficient Schwann cells to properly myelinate underlying axons is affected at the early stages of myelination, which is in line with the early onset of the neuropathy reported in CMT4C patients [69–71]. However, its exact role in myelin biology remains to be determined. Recent data demonstrated that SH3TC2 localizes at the plasma membrane and in endocytic vesicles [72–74] and that it interacts with the small GTPase Rab11, which is known to regulate the recycling of internalized membranes and receptors back to the plasma membrane. Further protein binding studies and transferrin receptor trafficking assays revealed that SH3TC2 together with Rab11 indeed affect the dynamics of endocytic recycling [75].

## 3. Cellular and Molecular Bases of Nerve Regeneration and Plasticity in CMT Neuropathies

The discovery of many genes involved in CMT disease has provided a unique opportunity to understand the critical molecular pathways involved in peripheral axon stability and length-dependent peripheral nerve disease [17, 18]. An important concept in peripheral neuropathy is that many types are characteristically length dependent; that is, the longest axons in the body are affected first and most profoundly. The length-dependent distribution supports the concept that the major site of pathology is in the axon itself, rather than the cell body. Furthermore, it suggests that shorter axons are either less susceptible or better able to compensate for certain insults that are longer axons, leading to the degeneration of the distal regions of the longest axons first [76]. Peripheral neuropathies presenting a distal nonterminal axonopathy represent the most common nerve diseases. Their long-term outcome depends on the balance of two processes: the degree or rate of axonal degeneration and the ability of the nascent axon tips to regenerate efficiently. One strategy to alter these processes would be to improve the efficiency of regeneration by using trophic factors such as neurotrophins [77], moving them from bench-to bedside. Prolonged denervation could lead to decreased regeneration capacity because of reduction in the expression of regeneration-associated Schwann cell molecules, such as neurotrophic factors and receptors. Therefore, Schwann cells might remain in a growth-supportive mode for prolonged periods or they have to be transformed into a competent premyelinating state to initiate and complete myelination. The functional significance of regeneration is to allow reinnervation of target organs and restitution of their corresponding functions. The materials for axonal growth are mainly provided by the cell body via axonal transport [78, 79], but more recently the contribution of local axonal synthesis and degradation of proteins has been identified [80, 81]. Increased energy demands on the neuron to propagate action potentials, and decreased trophic factor support from denervated Schwann cells or muscle are other potential mechanisms that may also contribute to axonal degeneration in demyelinating neuropathies (reviewed in [16]). An unanswered question with respect to all CMT1 forms is why mutant Schwann cells fail to support axonal function and survival [37]. An important direction is the development of therapeutic strategies that enhance axonal regeneration and promote selective target reinnervation; in addition, modulation of the central nervous system reorganization to improve functional recovery but also diminishing undesirable consequences has been proposed as well [82].

## 4. CMT Pharmacological and Biological Therapies

CMT disease course and severity vary according to CMT type, causative gene, and mutation change, but considerable phenotypic variability may occur also within the same CMT type. Understanding the molecular pathogenesis of inherited neuropathies is essential for the development of rational therapies (Figure 1). While much remains to be learned, it is clear that most are caused by the expression of a mutant allele(s) in myelinating Schwann cells or neurons. For recessive neuropathies, in principle it is possible to “replace” the defective gene by introducing a normal version. For dominant neuropathies, the situation is even more complex, as these are likely to be caused by a toxic gain of function that is not necessarily related to the normal function of the gene product. Nevertheless, for dominant demyelinating neuropathies caused by altered gene dosage, reestablishing the normal level of gene expression might be of benefit. The pathogenic mechanism in CMT1A duplication is attributed to an excess gene copy number of *PMP22*, leading to protein overexpression [28], and factors that modify the expression levels of *PMP22* might potentially be effective for treatment. A proof of concept that demyelination can be reversed by normalizing expression of *PMP22* was provided through a transgenic mouse model [83]. Research is focused on developing new treatment strategies to target the regulation of *PMP22* gene dosage. Two compounds that have been shown to alter *PMP22* mRNA levels in rodents are ascorbic acid and progesterone and progesterone antagonists.

**Ascorbic acid** reduced the severity of neuropathy in transgenic mice overexpressing *PMP22*, an animal model of human CMT1A, compared with untreated mice [84]. Ascorbic acid promotes myelination *in vitro* and decreases *PMP22 *mRNA levels through a cAMP-mediated pathway [84, 85]. Evidence of efficacy of ascorbic acid in the animal model prompted initiation of randomized controlled trials to test the efficacy of ascorbic acid in patients with CMT1A. Results from a phase 3, multicentre, placebo-controlled, double-blind randomized trial to assess the efficacy and tolerability of chronic ascorbic acid treatment in patients with CMT1A in Italy and the UK have been just published [86]. Unfortunately, ascorbic acid supplementation had no significant effect on neuropathy compared to placebo after 2 years followup, suggesting that no evidence is available to support treatment with ascorbic acid in adults with CMT1A.

It is known that progesterone and derivates are able to increase *MPZ* and *PMP22* gene expression *in vitro* [87]. In order to test if progesterone can modulate the progressive neuropathy caused by moderate overexpression of *PMP22,* Sereda and collaborators [88] administrated daily subcutaneous injections of progesterone and the **progesterone antagonist**, onapristone, to a transgenic rat model of CMT1A during 7 weeks and showed that onapristone reduced *PMP22 *mRNA by 15%, resulting in clinical and neuropathological improvement. A long-term study reaffirmed these results and shed light to the axonal degeneration process seen in CMT1A patients, by reducing progressive muscle atrophy and preventing axonal loss without altering myelin sheath thickness [89]. Unfortunately, onapristone and currently available progesterone antagonists are too toxic to be safely administered to CMT1A patients, so further research is ongoing to develop suitable compounds for future clinical trials. To accomplish this goal, the Charcot-Marie-Tooth Association (CMTA) has established the Strategy to Accelerate Research (STAR) to specifically fund CMT-related research (http://www.cmtausa.org/). High-throughput screens are trying to identify compounds that are already FDA approved, so it could accelerate the drug development process and start phase III clinical trials in 3–5 years.

Another interesting and promising molecule is **curcumin**, which plays a role stimulating the translocation of misfolded proteins from the endoplasmic reticulum to the plasma membrane, thereby reducing cytotoxicity of the mutant proteins. This mechanism might be helpful for selected CMT1A and CMT1B forms, in which various *PMP22 *and *MPZ *mutations are known to cause intracellular accumulation of mutant proteins. Oral curcumin mitigates the clinical and neuropathologic phenotype of *Trembler*-*J* mouse model of CMT1A, inhibiting Schwann cell apoptosis and increasing axonal caliber and myelin thickness. Furthermore, this positive clinical response to curcumin occurs in a dose-dependent manner and is reversed after withdrawal of treatment without side effects. Recent cell-based studies showed that mutant P0 could accumulate in the ER and induce apoptosis. This aggregation-induced apoptosis was abrogated by pretreatment with curcumin [90]. These findings suggest a potential therapeutic role of curcumin in selected forms of inherited peripheral neuropathies. There is also evidence that *MPZ* mutations with ER-retention of the mutated protein cause UPR activation rather than apoptosis [38] so this mechanism could also be relevant as a therapeutic target.

Regarding axonal CMT, in a transgenic mouse model for mutant *HSBP1*-induced CMT2 and distal HMN type 2B [91], mutant HSBP1, also known as HSP27, leads to severe axonal transport defects induced by a decrease in acetylated tubulin abundance in peripheral nerves. The phenotype was partially restored and the axonal transport defects were rescued when mice were treated with **HDAC6 inhibitors**. Histone deacetylase 6 (HDAC6) is the major enzyme with *α*-tubulin deacetylating activity. For this study the authors used a nonspecific inhibitor, trichostatin A (TSA), and two highly selective inhibitors: tubacin and tubastatin A, which resulted to be more effective compared to TSA when axonal transport and CMT phenotype were assessed. As some HDAC6 inhibitors have entered into clinical trials for cancer treatment and other neurological disorders such as Friedreich's ataxia [92], it is rationale to think that the same drug approach could be tested in CMT patients.

Schwann cell pathology damages the delicate myelin-axon interaction and can lead to axonal degeneration [6], but pronounced axonal pathology has also been observed even in genetic models in which axons are associated with normal appearing myelin sheaths. Thus, a therapeutic approach focusing on preventing this intimate connection could be providing **trophic factor support** to degenerating axons, which may be useful for a number of CMT neuropathies, either primary myelinopathies or primary axonopathies. The main conceptual problem with this approach is the diversity of trophic factors and PNS neurons, especially because different kinds of neurons respond to different trophic factors [16]. Only neurons with the proper receptors typically respond to a given growth factor. Receptor-mediated, retrograde axonal transport delivers the trophic factor to neuronal cell bodies, where they act. Axonal elongation requires an adequate substrate of trophins and tropic factors, provided by reactive Schwann cells and the extracellular matrix within the degenerated nerve [93]. Another potentially approach involves the manipulation of Schwann cell-axonal signal transduction pathways. Neuregulin-1 enhances axonal regeneration [94] by acting on Schwann cells as PNS neurons do not express neuregulin receptors. Axons express neuregulin-1 type III (Nrg1-III) on their surface, which binds to ErbB receptors on Schwann cells as part of a process that initiates myelination. Therefore, Nrg1-III acts as a juxtacrine signal. Nrg1-III binds to ErbB3 and promotes ErbB2 phosphorylation of tyrosine residues in the cytoplasmic domain of both ErbB2 and ErbB3 receptors [95]. Activation of the ErbB receptors leads to signaling through multiple signaling cascades including PI3K/Akt, Erk1/2, JNK, and FAK [96]. Three families of trophic factors are particularly important for PNS neurons: the neurotrophin family, the glial-derived neurotrophic factor (GDNF), and the ciliary neurotrophic factor (CNTF) family of cytokines. Sahenk and collaborators studied the ability of mutant Schwann cells to respond to exogenous neurotrophin-3 (NT-3) in two CMT1A animal models [97]. NT-3-treated animals presented improved nerve regeneration and the associated myelination process. Furthermore, at the early stages of regeneration-associated myelination, NT-3 stabilizes the axonal cytoskeleton locally by inducing neurofilament phosphorylation when axon sprouts become enwrapped by Schwann cells [77]. In contrast, BDNF, which belongs to the neurotrophin family but is not part of the Schwann cell survival loop [98], showed no effect upon axonal growth or cytoskeletal neurofilament pathology. Unfortunately, despite the promising results in animal studies, no studies have been successful in humans, probably due to a poor delivery and short half-lives of the trophic and growth factors. Targeting the correct combination of trophic factors to neurons or Schwann cells at the optimal time may be necessary to achieve meaningful results. A combination of trophic factors or engineered “pan-neurotrophic factors” [99] might be more beneficial than a single factor.

The short half-life of most neurotrophic factors would require either multiple administrations or a continuous infusion of the therapeutic molecules in order to achieve an adequate and effective local concentration. Knowing the molecular basis of inherited diseases prompts immediate consideration of **gene therapy**. Gene therapy can be defined as a strategy to transfer biologically relevant genetic material (usually mutant genes or genes delivering trophic and growth factors) into affected cells in the body to treat disease. For instance, delivering neurotrophic factors to the healing nerve ends, favoring survival and regeneration of both sensory and motor axons, could, ultimately, allow the recovery of nervous functions [100]. Viral vectors and plasmid DNA have been widely used for treating human disease models and patients. Expression of the gene might be modulated by the introduction of regulatory elements for the controlled or tissue-specific expression of the desired molecule. Until now, three major classes of viral vectors, based on adenovirus, adeno-associated virus (AAV), and herpes simplex virus (HSV), have been exploited to target the PNS. Although not originally neurotropic, adenoviral and AAV vectors are able to transduce spinal sensory and motor neurons after either intramuscular or intraneural injections. When injected at the site of a nerve injury, these vectors are retrogradely transported to motor neuron cell bodies and can thus be exploited to deliver therapeutic genes along the route of the nerve. However, the strong absorption of both adenoviral and AAV vectors to skeletal muscle fibers might represent a limitation for efficient neuronal transduction and retrograde transport [101]. A recent study in mice has characterized the tropism and transduction efficiency of different AAV pseudotypes after sciatic nerve injection. Among the pseudotypes tested, AAV2/1 transduced both Schwann cells and neurons, AAV2/2 infected only sensory neurons, and AAV2/8 preferentially transduced Schwann cells, proving the utility of AAVs as gene therapy vectors [102]. A few approaches have used plasmids as vehicles to deliver therapeutic genes to peripheral nerves. In these cases, the skeletal muscle has been the preferred site for delivery and expression of the transgene. For instance, intramuscular delivery of a plasmid encoding for vascular endothelial growth factor (VEGF) showed a protective role against myelin wasting and axonal loss [103]. Viral vectors have been modified so that they are unable to cause disease. Unfortunately, they have caused immunologic reactions, which currently limit their use. In contrast, plasmid DNA is nonimmunogenic, but it is characterized by poor delivery efficiency, and proteins made from it have only been produced in target organs for a short time. Progress in this area demands more sophisticated delivery systems and more knowledge of the molecular pathogenesis of neuropathies.

Schwann cells, and their basal lamina, represent the key component of nerve regeneration, as they serve as scaffolds for the regenerating axons, which grow through the empty basal lamina tubes. Schwann cells, however, have limited clinical applications since the culture of an adequate quantity of cells to achieve optimal conditions for transplantation in nerve conduits is time consuming and requires particular care for *in vitro *expansion and a constant input of growth factors. Moreover, Schwann cells are not easily accessible without nerve biopsy and bear the need to sacrifice an autologous nerve, with the related complications. Due to all these difficulties, the field of **stem cell therapy** for peripheral neuropathies has been explored. Adult stem cells show ability to differentiate into neuroprogenitor-type cells [104, 105]. Stem cells could be differentiating into neurons, which will generate new axons to contact their targets or Schwann cells enwrapping demyelinating axons and secreting trophic factors. Bone-marrow-derived mesenchymal stem cells (MSCs) can be induced to differentiate into Schwann cells [106], improving myelin formation and nerve regeneration *in vivo *after their transplantation into different models of peripheral nerve injury [107, 108]. Adipose tissue has been also indicated as a novel and promising source of multipotent cells (adipose-derived stem cells, ASCs), which can be differentiated into a neuronal phenotype [109, 110], and in terms of clinical use, they may be harvested by conventional liposuction procedure under local anaesthesia. The frequency of stem cells in adipose tissue is 100- to 1000-fold higher than that in bone marrow, which is a considerable advantage as it reduces the period of expansion of the stem cells prior to differentiation. Terenghi's group showed how ASCs could be differentiated towards a Schwann cell-like phenotype, expressing markers like S-100, glial fibrillary acidic protein (GFAP) and P75 neurotrophin receptor and enhancing neurite outgrowth in an *in vitro *co-culture model [111]. More recently, expression of myelin proteins P0 and PMP22 after differentiation of both ASC and MSC [112] and the neurotrophic potential shown *in vitro *from differentiated adipose-derived stem cells (dASCs) with a brief term *in vivo* study have been described [113].

## 5. Conclusions

Charcot-Marie-Tooth disease is a generalized disorder of motor and sensory peripheral nerves. Three major points deserve attention: (1) disease pathophysiology of both myelinopathies and axonopathies forms are the consequence of altered Schwann cell-axon communication; abnormal intercellular contact and signaling induce neurodegeneration and axonal loss, which ultimate produce muscular atrophy and weakness, (2) the primary cause is the genetic mutation in any of the more than 40 genes causing CMT or related neuropathies, and (3) a major therapeutic target is the regeneration of cell viability based on improving myelin and axon interaction. Therapies promoting plasticity changes in axons and Schwann cells require not only new therapeutic drug, gene, or cell approaches but also proper delivery systems targeted to the pathological cellular structures.
